# Emotional Analysis Model for Social Hot Topics of Professional Migrant Workers

**DOI:** 10.1155/2022/3812055

**Published:** 2022-01-31

**Authors:** Gefeng Pang, Anze Bao

**Affiliations:** School of Economics and Business Administration, Chongqing University, Chongqing 400030, China

## Abstract

Text makes up a large portion of network data because it is the vehicle for people's direct expression of emotions and opinions. How to analyze and mine these emotional text data has become a hot topic of concern in academia and industry in recent years. The online LDA (Latent Dirichlet Allocation) model is used in this paper to train the social hot topic data of professional migrant workers on the same time slice, and the subtopic evolution and intensity are obtained. The topic development is divided into four categories, and the classification model is created using SVM (Support Vector Machine). Instead of decision makers, a virtual human with sensibility and rationality is built using a hierarchical emotional cognitive model to solve multiobjective optimization problems interactively. It analyzes human body structure and emotional signals, and then combines them with visual and physiological signals to create multimodal emotional data. An example is used to demonstrate the effectiveness of the proposed model.

## 1. Introduction

Entrepreneurship of professional migrant workers can effectively alleviate social employment pressure, narrow the income gap between urban and rural areas, and promote new urbanization. In recent years, professional migrant workers' entrepreneurship has been paid more and more attention by the government and academia. However, at present, most professional migrant workers' new ventures are in a development dilemma due to lack of competitive advantages in the fierce market competition [1]. With the rapid spread of the Internet, its social effects are also rapidly expanding. These energies are positive and negative, while events with negative energy are more likely to cause large-scale discussions. At this time, local problems may become public topics in the country, causing huge social panic, and sometimes even requiring government intervention [2, 3]. The process of urbanization and the innovation of social system have increased farmers' enthusiasm to move to cities. They are eager to gain economic benefits in cities, but they are also eager to integrate into urban life, but the reality is not as they wish.

To deal with public opinion of professional migrant workers' social hotspots, it is necessary to improve the predictability of hotspots, which necessitates the analysis of professional migrant workers' social hotspots and trend prediction. The monitoring of professional migrant workers' social hot-spot public opinion requires the use of Weibo's hot-spot detection. It is necessary to understand the development trend of hot-spot in order to make the right decision and ensure the development of Weibo platform in the direction of health, freedom, and vitality in order to correctly guide public opinion and curb online rumors [4]. Different emotional themes focus on different aspects of emotional content in a subjective text and are also related to different emotional words in the text. As a result, the distribution of text themes can reflect the text's overall semantic structure. However, because the emotional content of the text is treated equally to other content in the theme modeling process, and the semantic strength of the emotional theme is determined by the proportion of emotional words in the text, it is still insufficient in highlighting the emotional semantics [5, 6]. Furthermore, traditional theme modeling ignores semantic relationship patterns such as text sequence and word context, instead treating the text as a word bag and determining the theme solely based on the word co-occurrence relationship, which limits the text representation ability.

This topic focuses on emotional analysis and topic trend prediction of social hot topics for professional migrant workers, which refers to the trend prediction of topic state change, that is, to predict the change of topic state in the next period of time [7]. Through human-computer interaction and fuzzy cognitive evaluation, the quantitative relationship among personality characteristics, mood state, and emotional state is described. On this basis, a virtual human integrating sensibility and rationality is proposed, and then a multiobjective decision-making problem-solving method based on hierarchical emotional cognitive model [8] is given. The visual signals are linearly fused with four physiological signals to obtain four groups of multimodal information [9]. The principle based on feedback information and the recognition rate of multimodal information to emotional state is introduced to design the weight determination method. On this basis, decision-level weighted fusion is introduced, and an emotion recognition model based on multimodal signal decision-level weighted fusion is established.

The innovation of this paper: This topic focuses on the emotional analysis of social hot topics of professional migrant workers on the Weibo platform. Based on the existing historical data, this paper deeply analyzes the characteristics of users and texts, extracts a series of characteristics such as emotions and users from them, and constructs a regression model combining with the time series model to describe the development trend of hot topics in the near future, and finally predicts the development trend of hot topics, and helps to realize the early warning of the development of hot topics.

## 2. Related Work

A research hotspot is using emotion classification technology to solve practical problems in social hot topics of professional migrant workers. The method of statistical emotion words is used in literature [10] to calculate emotions under fixed topics, which can be applied to trend prediction and yields good results. Hong et al. [11] calculate the emotional time series using the ratio of Weibo number to positive and negative emotional words obtained from Twitter data every day, and then use a self-organizing fuzzy neural network to predict the Dow Jones Industrial Average. To predict the movie box office, the emotional ratio is calculated using emotional analysis of social hot topic data, which is then combined with the movie box office using the Pearson correlation coefficient [12]. This paper uses statistics to conduct an emotional analysis on the topics of these mobile phones on Twitter, and then uses classified methods to predict users' satisfaction [13–15]. Zhou et al. [16] looked at opinion leader fans on Twitter, classified their emotional comments, and discovered that followers are influenced by their emotions, and that the change in this influence is consistent with reality. This study confirms that a user's emotions have an impact on his followers, particularly opinion leaders' emotional changes, and uses this relationship to predict user emotional changes.

The research on emotional modeling and application has been paid extensive attention [17, 18]. Especially, literature [19, 20] puts forward the emotional cognitive learning and decision-making model by applying Boltzmann selection mechanism, which takes emotional factors as internal influences and external environment as external influences, and realizes emotional cognitive learning and decision-making by calculating the internal and external influence probabilities after decision-making; Sosa and Rodríguez [21] realized the coordinated control of robot behavior on the basis of emotional cognitive learning and decision-making model. However, the application of emotional model was limited because it adopted symbolic cognitive state and decision-making behavior. In literature [22], the author proposed an improved emotional interaction model, but it still did not take into account the influence of individual personality characteristics on the emotional model, so it was still limited when it was used to solve decision-making problems. Khare and Bajaj [23] put forward a multimodal emotion recognition model based on visual signals and physiological signals, which combines facial expression features and ECG features in series at feature level to form multimodal features. Experiments show that the recognition rate of multimodal features is higher than that of one modal feature. Schmidt et al. [24] put forward the multistream fusion hidden Markov model for emotion recognition. The multistream fusion hidden Markov model is the generalization of the two-stream fusion hidden Markov model, which is a general model-level modal fusion method. Mohammed and Karim [25] use the principle of equal weight to weight and fuse the classification results of audio, video, and text at the decision level, which is equivalent to unweighted fusion at this time. Tao [26] uses grid traversal (0,1) to get the optimal weight, and at the decision level, the classification results of facial expression signals and ECG (electrocardiogram) signals are weighted and fused to get the final recognition result.

Based on the above research, aiming at the characteristics of multimodal information, linear fusion is introduced at the feature level to obtain multimodal features, and the expressive force of multimodal features on emotional state is analyzed. Combining with the principle based on feedback information, the weight determination method is designed. Linear weighted fusion is introduced at the decision level, and an emotion recognition model based on multimodal information two-level fusion is established. Therefore, combining with the characteristics of human multimodal emotional information, it is of great significance to study the weighted fusion of feature level and decision level to improve the model recognition rate.

## 3. Research Method

### 3.1. Research on Long-Term Topic Prediction Based on Subtopic Separation

**Figure 1:**
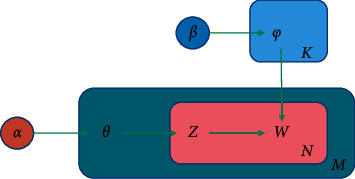
LDA model diagram.

LDA (Latent Dirichlet Allocation) topic model belongs to the document generation model, which assumes that the document has multiple hidden topics, all of which can be composed of the word distribution in the fixed vocabulary [27]. After the topic was put forward, it was widely used, especially in the direction of text clustering and similarity calculation.  Figure 1 is the LDA model diagram.

Assume that the rectangular square at the top of LDA graph model is the theme, and *K* is the number of themes. The letter *M* in the figure represents the number of all documents in the model, while *N* is the number of words in the documents and *Z* is the number of words in the word list.

The inner and outer boxes in the figure also have specific meanings, in which the outer box is a document, while the inner box represents the choice of topics and words in the document. The superparameter in the graph is defined as follows:

*α* represents document-topic distribution, and *β* represents topic-word distribution, both of which are fixed values, which are specified by users in advance. *W* in the graph is observable data, which is a *M* × *N*-dimensional matrix, and *W*_*ij*_ represents the word *j* in document *i*; Document-topic distribution *θ* and topic-word distribution *ϕ* are both implicit variables.

The process of using LDA model to generate documents is as follows:

1. Selecting word distribution *ϕ*_*k*_, *k* ∈ {1, ⋯, *K*} of topic *k* from Dir (*β*) of parameter *β*
2. Select the theme distribution *θ*_*i*_ of document *i* from Dir (*α*) of parameter *α*, *i* ∈ {1, ⋯, *M*}
3. For the word at the *j* position in document *i*, *i* ∈ {1, ⋯, *M*}, *j* ∈ {1, ⋯, *N*}:

1. Generating the theme *Z*_*ij*_ of the word *j* in the document *i* according to the polynomial distribution with the parameter *θ*_*i*_
2. According to the polynomial distribution with parameter *ϕ*_*Z*_*ij*__, the word *W*_ij_ of word *j* in document *i* is generated

A topic will inevitably change with time, but no matter how it changes, the new data will still carry the information of the old data, that is, the topic is persistent and stable. However, the training process of LDA model is to train all documents directly and generate various topics at the same time, which cannot deal with online new texts. Therefore, online LDA model should be born. In online LDA model, the distribution of topic words in time slice *t* obeys the following Dirichlet distribution:

(1) $$\begin{array}{c} \phi_{k}^{t}∼\text{Dirichlet}\left(\beta_{k}^{t}\right)∼\text{Dirichlet}\left(w\beta_{k}^{t-1}\right). \end{array}$$

Among them, the matrix *β*_*k*_^*t*−1^ retains the words of the previous time slice, and *w* is a weight vector indicating the proportion of historical words retained.

Set the theme intensity of this article, that is, the document with clear theme tendency is regarded as high intensity, otherwise it is low. Calculate the intensity of each Weibo, finally get the total intensity of Weibo under the time slice, and calculate the theme intensity according to

(2) $$\begin{array}{c} T_{S}\left(Z_{K}\right)=\frac{\sum_{n=1}^{N}w_{n}^{\theta_{n,k}}}{\sum_{n=1}^{N}w_{n}}. \end{array}$$

Among them, *θ*_*n*,*k*_ represents the estimated value of the distribution of the *m*-th Weibo on the *k*-th subtopic, and *w*_*n*_ represents the document weight. When the social hotspot is only related to this subtopic, the weight is set to 1. When social hotspots discuss multiple events at once, as shown in LDA, that is, when words with multiple topics appear in this social hot topic of professional migrant workers, and the weight is measured by the maximum value, the weight is measured by the maximum value.

When the theme evolves, the words change with it. The so-called variation is the dissimilarity. KL distance is generally used to measure the similarity. For the topics of *d* − 1 time and *d* time, KL distance is

(3) $$\begin{array}{c} S_{\text{KL}}=\left(\phi_{k}^{d-1},\phi_{k}^{d}\right)=\frac{1}{2}\left(\sum_{i=1}^{V}\phi_{k,i}^{d-1}\log\frac{\phi_{k,i}^{d-1}}{\phi_{k,i}^{d}}\right), \end{array}$$

where *ϕ*_*k*_^*d*−1^, *ϕ*_*k*_^*d*^ is the distribution of the same subtopic on the previous day and the next day, respectively, and the calculation result indicates the similarity between the two topics, then the topic variation rate is as shown in formula (4), which is

(4) $$\begin{array}{c} V=1-S_{\text{KL}}\left(\phi_{k}^{d-1},\phi_{k}^{d}\right). \end{array}$$

**Figure 2:**
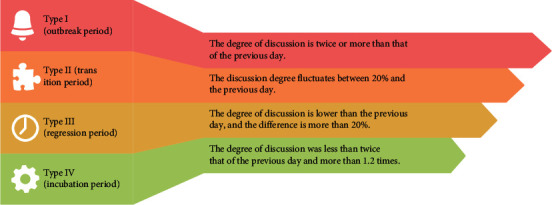
Classification table of long-term topic popularity value.

Considering that the ups and downs cannot reflect the overall change of the trend, according to the speed of topic trend change, the topic discussion degree is divided into four categories, and forecasts them separately. The specific division according to numerical values is shown in Figure 2.

These four categories are classified in this chapter based on subtopic intensity and subtopic variation rate, and the subtopic intensity and subtopic variation rate, as well as the classification results, are added as two features to each time slice, and the prediction is made using the GBDT method (gradient boosting decision tree).

### 3.2. Hierarchical Emotional Cognitive Model

**Figure 3:**
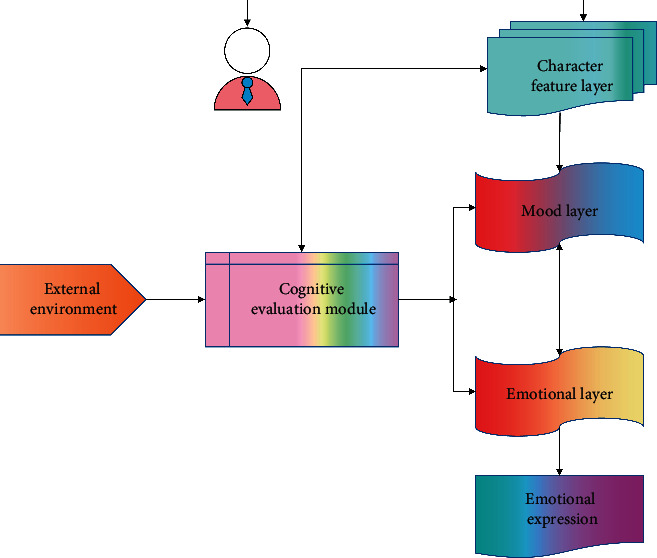
Hierarchical emotional cognitive model structure.

Establish a hierarchical emotional cognitive model, which includes a personality characteristic layer, a mood state layer, an emotional state layer, and a cognitive evaluation module, as shown in Figure 3.

The personality layer realizes the acquisition of personality data through human-computer interaction, and influences the change process of the lower mood state through mapping and personality parameters; the cognitive module quantifies and evaluates the influence of external stimulus signals on the emotional model, and then transmits it to the mood layer of the emotional model.

In the process of emotion renewal, mood layer and emotion layer interact with each other, and the stability of personality layer, mood layer, and emotion layer decreases in turn, while personality layer is the most stable and emotion layer is the most prone to change.

Personality is a relatively stable parameter that can be used to describe a person's personality and is influenced by both heredity and acquired environment. Personality is an important parameter in the emotion model because it directly reflects the personality of the individual. Because personality has a direct impact on the size of each component of mood state and the speed with which states transition, emotion modeling requires accurate data on individual personalities.

The definite character characteristic vector is

(5) $$\begin{array}{c} P_{p}=\left[P_{N},P_{E},P_{O},P_{C},P_{A}\right],\\ P_{X}\in \left[0,1\right],\\ X\in \left\{N,E,O,C,A\right\}. \end{array}$$

Among them, *P*_*N*_, *P*_*E*_, *P*_*O*_, *P*_*C*_, *P*_*A*_ represents neuroticism, extroversion, openness, conscientiousness, and agreeableness, respectively.

Mood level is used to represent people's mood state, which can be described by PAD (pleasure-arousal-dominance) model, where *P* represents the positive and negative characteristics of individual emotional state, *A* represents the individual's neurophysiological activation level, and *D* represents the individual's control over the situation and others. PAD model corresponds to a three-dimensional emotional space.

Define the mood state space as a three-dimensional PAD, and define the mood state vector as

(6) $$\begin{array}{c} M_{\text{ent}}=\left[m_{P},m_{A},m_{D}\right]. \end{array}$$

Among them *m*_*P*_,  *m*_*A*_,  *m*_*D*_ ∈ [−1,1], *M*_ent_=[0,0,0] corresponds to a calm state of mind.

In order to be suitable for multiobjective decision-making, two components, positive and negative characteristics of emotion and emotional intensity, are selected to represent the emotional state. The emotional state is defined as a two-dimensional vector:

(7) $$\begin{array}{c} E_{e}=\left[\chi ,q\right],\chi \left\{-1,0,1\right\},q\in \left(0,1\right). \end{array}$$

Among them *χ* represents the positive and negative characteristics of emotion, with a value of 1, 0, or 1, which respectively indicates that the current emotional state is negative, calm, and positive; *q* represents the current emotional intensity.

The cognitive evaluation of an emotion model for external stimulus signals should begin with the individual's intention and evaluation criteria for the evaluation subject, and then learn from the literature method [28]. Individuals' subjective wishes, preferences, and individual evaluation criteria determine their emotional expression, so the emotion model should pay attention to the design of wishes, preferences, and evaluation criteria for the cognition of stimulus signals.

The change of state of mind is influenced by personality characteristics and external stimuli, and at the same time, it will decay with time. Let *M*_*t*_ represent the state of mind corresponding to time *t*, then the updating process of state of mind space is as follows:

(8) $$\begin{array}{c} M_{t}=\varphi \left(M_{t-1}\right)+M_{P}+M\left(U\right) \end{array}$$

Here, *M*_*t*_,  *M*_*t*−1_ is the state of mind of the current moment and the previous moment respectively; *φ*(*M*_*t*−1_) is the mood state attenuation function; and *M*(*U*) is the influence of external stimuli on mood, which is calculated by fuzzy cognitive evaluation module.

Normal multiobjective decision-making problem can be described as

(9) $$\begin{array}{c} \max\;M=\left\{f_{1}\left(x\right),f_{2}\left(x\right),\ldots ,f_{l}\left(x\right)\right\}\\ s\text{.t}\left\{\begin{array}{cc} g_{i}\left(x\right)\leq 0, & i=1,2,\ldots ,m,\\ X={\left(x_{1},x_{2},\ldots ,x_{n}\right)}^{T}, & x_{*}\in R, \end{array}\right) \end{array}$$

where *g*(*x*) is the constraint function.

**Figure 4:**
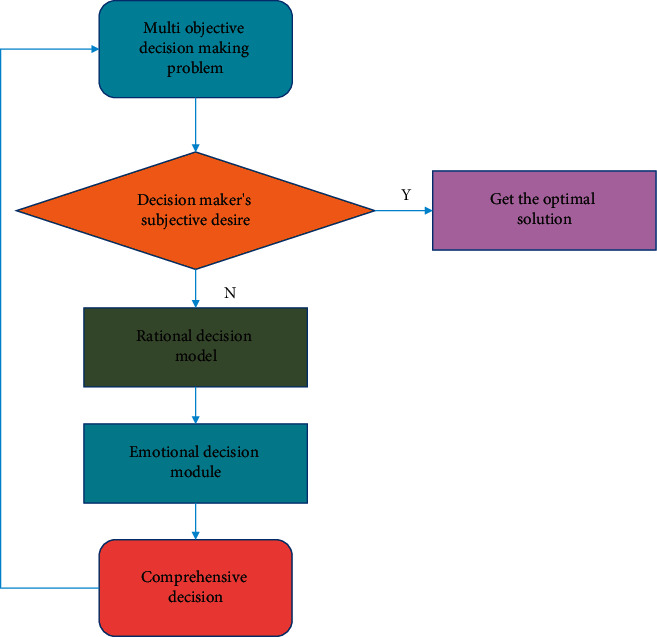
Cognitive decision-making process.

Figure 4 shows an emotional interactive decision-making algorithm based on hierarchical emotional cognitive model. This algorithm takes goal achievement degree, priority of objective function and overall coordination degree as emotional cognitive evaluation factors, and combines rational decision-making with emotional decision-making to build a virtual human. After obtaining the cognitive model of decision-making experts through interaction, it can realize automatic emotional decision-making by computer.

Cognitive decision-making model consists of rational decision-making module and emotional decision-making module, which respectively give the adjustment range of goal attainment. The two parts of adjustment range of goal attainment represent external objective requirements and internal subjective desires, respectively.

### 3.3. Emotion Recognition Based on Multimodal Information Feature Level and Decision Level Fusion

Emotion can cause many changes such as external changes of facial expression and internal changes of human physiology at the same time, so it is imperative to study emotion recognition based on multimodal information [29]. Facial expression recognition based on visual signals, considering the dynamic feature information of facial expression changes, and using the temporal and spatial information of facial expression changes at the same time, can reflect the essence of facial expression changes more truly and has stronger practical applicability.

The process of expressing human emotions is a process in which various modal information complement each other comprehensively. Single-modal features can only show part of the attribute information of the object. In order to describe the target object more accurately, the integration of multimodal features is an inevitable trend. The emotional features involved in this chapter are visual signal features and four physiological signal features. After feature extraction [30–32], the visual signal features and four physiological signal features are combined in series to form four multimodal features.

SVM (Support Vector Machine) is a new learning algorithm developed on the basis of statistical learning theory. Support vector machine classifier has a good effect on small sample classification problem, and it is not necessary to take enough sample number as the theoretical condition. For the above reasons, this method has been widely used in pattern recognition.

This chapter chooses one-to-one method to solve the multiclassification problem of expressions based on support vector machine. For *n* expression categories, design a binary support vector machine classifier for every two expressions, that is, design *n*(*n* − 1)/2 subsupport vector machine classifier, *SVM*_*ij*_, 1 ≤ *i* < *j* ≤ *k*. Among them, *i*, *j* is the expression category label.

In this chapter, the voting mechanism of multiple classifiers is adopted, and the complementary performance of multiple classifiers is used to improve the recognition effect. First, the recognition results of emotion state by emotion classifier based on multimodal signals are obtained. Then, for the experimental results of several subclassifiers, the final recognition results are obtained by weighted voting, and the specific calculation steps are as follows:

Get the weighting matrix of the multimodal signal, and then the weighting matrix *W*_*i*_(1 ≤ *i* ≤ 4) of the corresponding subclassifier is

(10) $$\begin{array}{c} W_{i}=\left[\begin{array}{ccc} p_{i1} & \cdots & 0\\ \vdots & \ddots & \vdots \\ 0 & \cdots & p_{im} \end{array}\right]. \end{array}$$

Let ${\overset{⟶}{C}}_{i}={\left(c_{i1},c_{\text{im}}\right)}^{T}\left(1\leq i\leq 4\right)$ be the result of the subclassifier, where $\left|{\overset{⟶}{C}}_{i}\right|=1,c_{\text{ij}}\in \left\{0,1\right\}\left(1\leq i\leq 4,1\leq j\leq m\right)$.

(11) $$\begin{array}{c} \overset{⟶}{C}=\sum_{i=1}^{4}W_{i}{\overset{⟶}{C}}_{i}=\sum_{i=1}^{4}\left[\begin{array}{ccc} p_{i1} & \cdots & 0\\ \vdots & \ddots & \vdots \\ 0 & \cdots & p_{im} \end{array}\right]\left[\begin{array}{c} c_{i1}\\ \vdots \\ c_{im} \end{array}\right]=\left[\begin{array}{c} \sum_{i=1}^{4}c_{i2}p_{i1}\\ \vdots \\ \sum_{i=1}^{4}c_{im}p_{im} \end{array}\right]. \end{array}$$

Based on the maximum rule, the *k*-type emotional state with the highest score is the final recognition result, as shown below:

(12) $$\begin{array}{c} \overset{m}{\underset{j=1}{\text{Max}}}\left\{\sum_{i=1}^{4}c_{ij}p_{ij}\right\}=\sum_{i=1}^{4}c_{ik}p_{ik}. \end{array}$$

**Figure 5:**
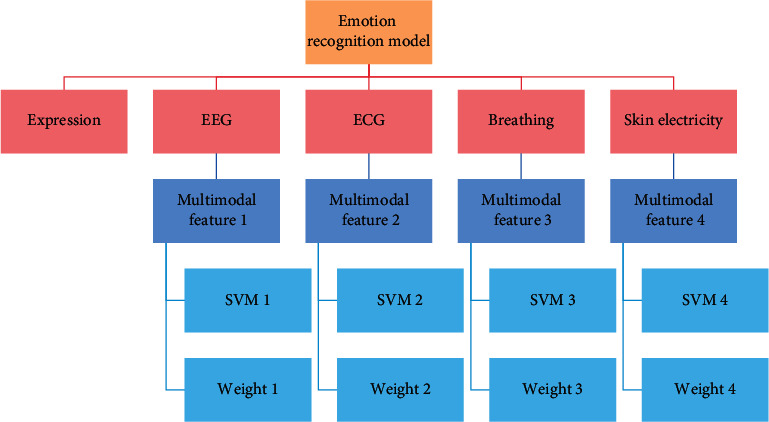
Emotion recognition model based on feature-level fusion and decision-level weighted fusion.

For the emotion recognition model based on multimodal signals, because many modal signals are related to the emotional state, analyze the physiological structure of human body and the characteristics of emotion signals, and optimize the selection of visual signals (facial image signals) and four physiological signals (EEG (electroencephalogram) signals, ECG signals, respiratory signals and skin signals) that can be used for emotion recognition, as shown in Figure 5.

Four subclassifiers based on support vector machine are established, and four subclassifiers are trained and tested by using four multimodal emotion features, and the emotion recognition results are obtained. Finally, the model is trained and tested with four multimodal emotional features, and the final recognition result is obtained.

## 4. Analysis and Discussion

### 4.1. Long-Term Topic Prediction Analysis

**Figure 6:**
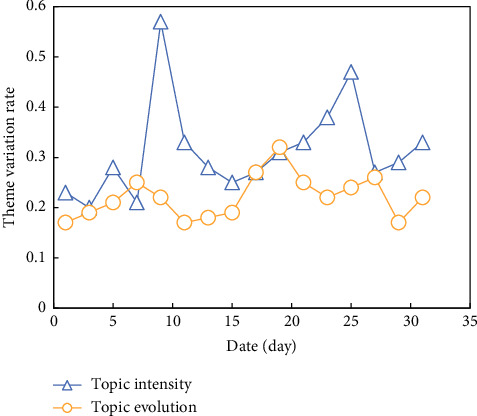
Topic intensity and topic evolution trend chart.

Online LDA model trains data, using all data of the same topic every time, and dividing the data into different documents for each topic according to the daily time slice, and numbering the documents from 0 according to the date sequence. Compare the topic evolution and topic intensity with the topic discussion degree, as shown in Figure 6.

It can be found that when the topic variation rate and intensity are high, the topic discussion degree is also greatly improved, which shows that the topic discussion degree has a great relationship with the topic variation rate and the topic intensity. Careful observation shows that the trend chart between the topic variation rate and the topic intensity has the following relationship:

1. The number of peaks is roughly the same, and they can all find the correspondence in the topic heat
2. Theme enhancement lags behind the peak of theme evolution diagram
3. The small peak of the topic is not closely related to the topic intensity and topic variation, but the big peak is closely related, and the peak of topic evolution is before the peak of the topic

SVM is widely used in classification and regression applications. In this section, SVM in sklearn Toolkit is used to classify forecast data into four categories, with the following characteristics:

1. Subtopic intensity in the first four days
2. Subtopic variation rate in the first four days
3. The historical heat of the first four days
4. Text features of the previous 4 days

**Figure 7:**
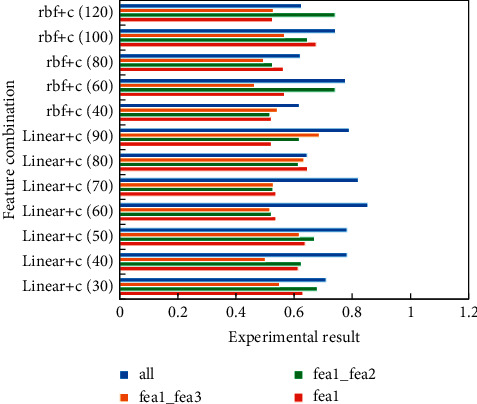
Comparison of experimental results with different characteristics.

After the features are normalized, SVM is used for training. SVM is very sensitive to the penalty factor *c*. gridsearchcv () in sklearn is used to automatically find the parameters by gridding.  Figure 7 shows the subjective and objective classification error rates corresponding to four groups of feature combinations under several representative *c* values.

Theme evolution and theme intensity features are recorded as fea1, text content features as fea2 and data features as fea3.

**Figure 8:**
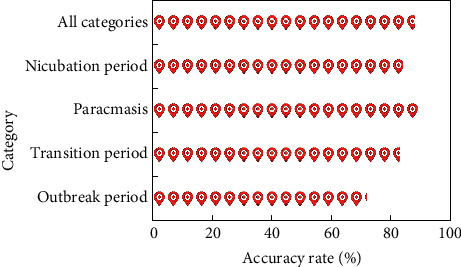
Four classification results.

After carefully adjusting the parameters again, the kernel function selects the linear kernel function, the parameter *c*=80, *g*=0.03. The final result is shown in Figure 8.

It can be found that the peak prediction rate is very high, which is very helpful for our future trend prediction, and the overall classification accuracy rate reaches 88%. In addition, it is found that these four stages usually occur in turn, so when the category is accurately predicted, this feature is added to the prediction feature to predict the data between two peaks.

**Figure 9:**
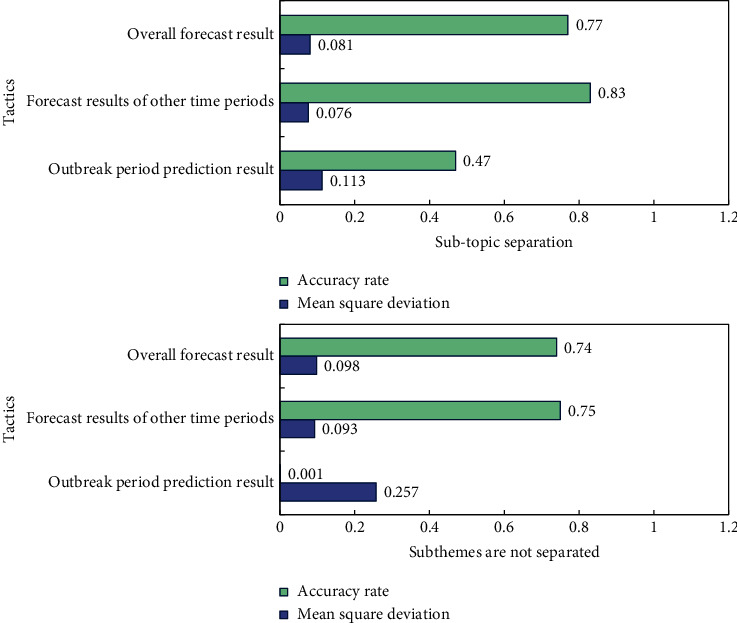
Trend prediction result.

In this section, four kinds of classification results are predicted in turn. Only the features of the previous day are used to predict the peak part, and all data before the arrival of the next peak are predicted by using the features of historical data of 4 days. If the calculation is less than 4 days from the peak, the previous features are filled with 0, and the GBDT regression method is used.  Figure 9 shows the trend prediction results.

From Figure 9, it can be seen that, after introducing online LDA model to separate subtopics, the mean square error dropped from 0.098 to 0.081, and the accuracy rate reached 0.77 from 0.74, both of which were better than the previous methods. Moreover, none of the previous forecast periods were accurate. After separation, most outbreak periods can be predicted accurately, but there is still a certain gap between the predicted values.

### 4.2. Cognitive Analysis

Firstly, the corresponding emotional cognitive model is established. By evaluating the decision makers' personality, the decision makers' personality parameters are $P_{P}=\left[\begin{array}{ccccc} 0.33 & 0.21 & 0.56 & 0.72 & 0.83 \end{array}\right]$ and the influence component of mood is $M_{P}=\left[\begin{array}{ccc} 0.56 & 0.12 & 0.14 \end{array}\right]$.

When the optimal solution is *x*^*∗*0^=(2.1, 3.1, 3.6), the goal achievement degree *μ*^0^=(0,0,0) and the overall coordination degree *λ*^0^=0.6 are calculated. Then, the multiobjective decision-making is solved based on this emotional cognitive model.

**Figure 10:**
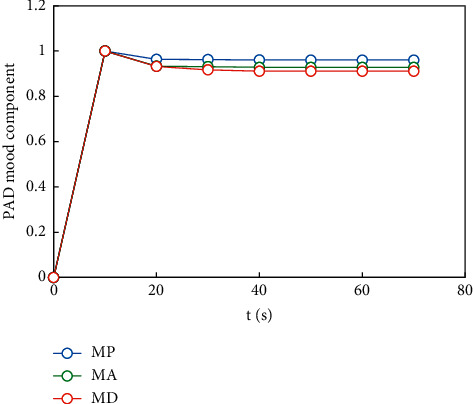
The transition process of each component of decision-making mood state of objective 1.

**Figure 11:**
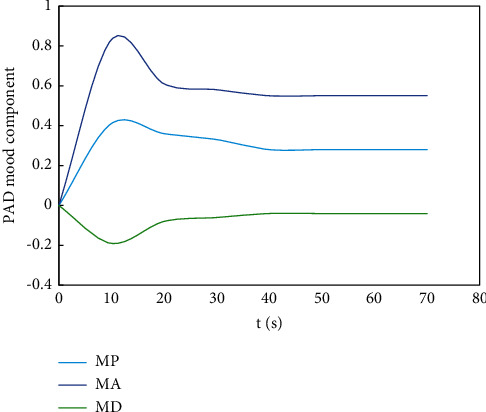
The transition process of each component of decision-making mood state of objective 2.

**Figure 12:**
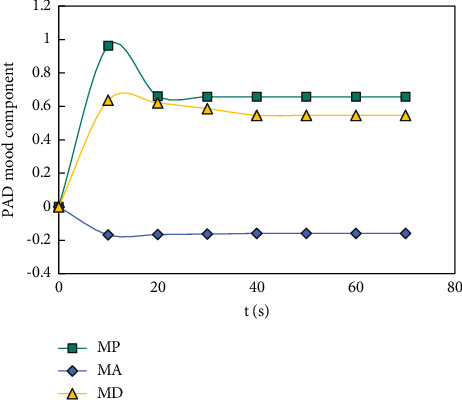
The transition process of each component of decision-making mood state of objective 3. At this point, Δ*μ*^3^=(−0.186, 0.028, −0.034), the optimal solution of the third interactive decision is: *x*^*∗*3^=(10.036, 8.875, 7.518), *f*(*x*^*∗*3^)=(288.24, 241.28, 201.63).

On this basis, the third emotional cognitive decision was made, and the result obtained by the rational decision module was Δ*μ*_1_^3^=(0.233, −0.167, −0.332). The component transitions are shown in Figures 10–12.

Goal attainment: *μ*^3^=(0.778, 0.639, 0.507), overall achievement: $\lambda^{3}=0.778,s=\sqrt{\sum_{i=1}^{3}\left(\mu^{3}\left(i\right)-\mu^{2}\left(i\right)\right)\wedge 2}=0.063<0.1$, and satisfied *μ*(*f*_1_(*x*^*∗*3^)) > *μ*(*f*_2_(*x*^*∗*3^)) > *μ*(*f*_3_(*x*^*∗*3^)), Therefore, *x*^*∗*3^ is the optimal solution to satisfy the subjective desire of decision makers.

### 4.3. Emotion Recognition

In this section, the experimental hardware devices are mostly desktop computers. The hardware configuration is as follows: inter (R) CORE (TM) i7-6700 CPU, 3.4 GHz, 4 GB memory, and 64 bit Windows 7 Ultimate operating system, which is primarily responsible for running various experimental tools and software, data processing, and result output. MATLAB and the EEG Lab toolbox are used to process a variety of physiological data, and the LibSVM software package is used to train and test the SVM classifier, with Python as the development environment.

The multimodal responses and self-emotional evaluations of 30 collected subjects were induced and recorded synchronously using 20 video clips from the database as stimuli. Emotional signals included peripheral/central nervous system physiological signals, image signals, audio signals, and eye tracking signals. The database's objects are of various ages, genders, races, cultures, and educational backgrounds, and the information coverage is extensive. Furthermore, the emotion-induced video is highly stimulating, the data collection environment is standard, and the emotion signals collected are of high quality.

First, the data set is divided into five types based on emotional tags using stratified random sampling. The training set is made up of a certain percentage of data samples extracted from each physiological signal's data set, while the test set is made up of the remaining data samples.

**Figure 13:**
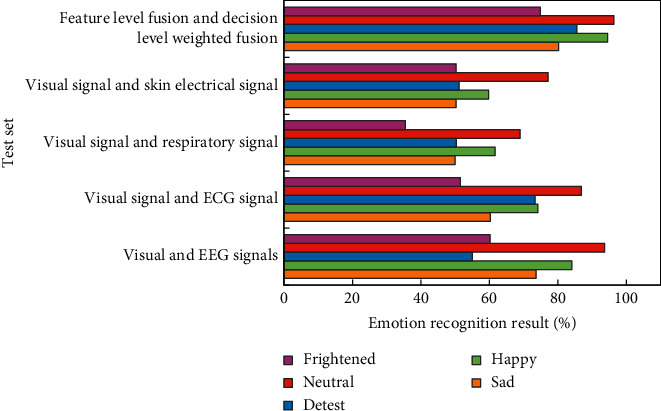
Emotion recognition results based on four multimodal emotion signals and two-level fusion.

The emotion recognition model is trained and tested by using four kinds of multimodal signal emotion features, respectively, and the corresponding emotion recognition results are shown in Figure 13.

It can be found from Figure 13 that the local recognition rate of four emotional states is based on the multimodal signal 1 composed of visual signals and EEG signals, while the highest recognition rate of one emotional state “disgust” is based on the multimodal signal 2 composed of visual signals and ECG signals. In addition, the expressive power of four multimodal signals to five emotional states is different.

The types of emotional signals are analyzed in this chapter's research, and a feature-level fusion method is proposed that is universal and can be used for any multimodal emotional signal. This paper investigates the expressive power of multimodal emotional signals in identifying emotional states and proposes a universal weight determination method based on multimodal emotional signals for identifying emotional states. A universal decision-level weighted fusion modeling method is proposed, which can be applied to any multichannel emotional data. The modeling method based on two-level fusion can fully exploit the benefits of each channel's emotional information, resulting in a higher model recognition rate.

## 5. Conclusion

Knowing the information trends of social events on the Weibo platform in real time, as well as tracking and predicting the social hot topics of professional migrant workers on a continuous basis, allows the government and enterprises to grasp public opinion trends in real time and guide public opinion, which is of great social importance to both the government and businesses. In this paper, the Weibo data is trained using an online LDA model on the same time slice, and the subtopic evolution and intensity are calculated. The development of the topic is divided into four categories. The classification model is built using SVM, and the data between two peaks is predicted using a short-term prediction model. The experimental results show that this method's topic popularity classification accuracy is 88 percent. A multiobjective emotional cognitive decision-making method is presented in this paper. This study lays the groundwork for implementing computer-assisted emotional decision-making in complex multiobjective problems. The classification results of four physiological signals are fused for decision-making according to the maximum rule, giving full play to the benefits of various physiological signals and thus improving the model recognition rate.

In this paper, an online LDA model is used to analyze the evolution of subtopics in long-term topics. However, due to the model's limitations, only the same number of subtopics can be fixed every day, which will need to be improved in future research. Various external factors, such as news media reports, public opinion climate, and so on, will influence the topic's trend.

## Data Availability

The raw data supporting the conclusions of this article will be made available by the authors, without undue reservation.
